# Development and validation of a novel phonomimetic bioreactor

**DOI:** 10.1371/journal.pone.0213788

**Published:** 2019-03-14

**Authors:** Andrijana Kirsch, David Hortobagyi, Theresa Stachl, Michael Karbiener, Tanja Grossmann, Claus Gerstenberger, Markus Gugatschka

**Affiliations:** Department of Phoniatrics, ENT University Hospital Graz, Medical University of Graz, Graz, Austria; German Cancer Research Center (DKFZ), GERMANY

## Abstract

Vocal fold fibroblasts (VFF) constitute the main cell type of the vocal fold’s lamina propria, produce the extracellular matrix and thereby determine the tissue characteristics. To study VFF behavior under *in vitro* conditions it is important to mimic the dynamic environment of the *in vivo* state. The aim of our study was to develop and validate a novel phonomimetic bioreactor system mainly based on commercially available components. The use of cell culture dishes with flexible silicone bottoms in combination with a suitable loudspeaker made it possible to expose the cells to various kinds of phonatory stimuli. The fundamental vibration characteristics of silicone membranes were investigated with and without cell culture medium by laser Doppler vibrometry. Human VFF were seeded in flexible-bottomed plates and placed in a custom-made housing containing a loudspeaker. After the cells were exposed to a predefined audio stimulation protocol, cell viability was assessed and gene as well as protein expression levels were compared to static controls. Laser Doppler vibrometry revealed that addition of cell culture medium changed the resonance frequencies of vibrating membranes. Gene expression of hyaluronan synthase 2, collagen III, fibronectin and TGFβ-1 was significantly upregulated in VFF exposed to vibration, compared to static control. Vibration also significantly upregulated collagen I gene and protein expression. We present a new type of phonomimetic bioreactor. Compared to previous models, our device is easy to assemble and cost-effective, yet can provide a wide spectrum of phonatory stimuli based on the entire dynamic range of the human voice. Gene expression data of VFF cultured in our phonomimetic bioreactor show a significant effect of vibration on ECM metabolism, which illustrates the efficacy of our device.

## Introduction

The exploration of molecular pathways in vocal fold (VF) biology and novel treatment strategies (e.g. laryngeal tissue engineering) is impeded by the lack of knowledge of cellular response to relevant mechanical stimuli, primarily vibration. The VFs are unique structures housed in the laryngeal skeleton enabling voice production (phonation). During phonation, the human VF tissues regularly experience vibrations greater than 120 Hz, a condition not seen in any other tissue[1]. The VFs are composed of a multi-layered squamous epithelium, the basement membrane zone, the *lamina propria* (LP) and the vocalis muscle. An intact LP is essential for physiological function and is mainly composed of vocal fold fibroblasts (VFF) and extracellular matrix (ECM) components (collagen fibres, hyaluronic acid etc.). It is divided in three distinct layers based on the histological composition of the ECM [2]. VFF maintain viscoelasticity for VF vibration by producing important ECM components. During phonation, the VFF experience complex tensile, shear, aerodynamic and contractile stresses [3]. It is generally accepted that extracellular and intracellular changes occur as a result of the vibration, which in turn alters gene and protein expression [4–6]. Injury to the VF due to vocal overuse or surgical procedures modify the behaviour of VFF, which may result in numerous VF pathologies (e.g. polyps, Reinke’s edema, VF scars) [7]. The resulting deterioration of the voice (dysphonia) reduces quality of life, leads to social withdrawal and affects the ability to work (e.g. teachers, call-center agents) [8].

Crucially, the special anatomical location, the sensitive microarchitecture and unique function of the human VF make it virtually impossible to explore VF pathophysiology on the cellular level. In addition, diagnosis and monitoring are still restricted to non-invasive visual (laryngo-stroboscopy) and perceptual (auditory) examinations. Thus, due to the lack of *in vivo* biological measures, many basic questions in laryngology remain unanswered. Although it is possible to cultivate VF cells *ex vivo*, the static nature of classical *in vitro* cell culture lacks the biophysical microenvironment of the native tissue.

At the same time, great hope is put in regenerative medicine and tissue engineering to provide treatment for some VF diseases, namely VF scarring and sulcus vocalis [9,10]; the recent development of a fully bio-engineered, tri-layered laryngeal mucosa graft might overcome the burden of VF scarring by transplantation of a tissue-engineered graft [10]. However, previous studies of unphonated larynges have shown that biomechanical stimulation is a decisive factor in the development of the LP microstructure [11].

New *in vitro* models to study VF biology are therefore highly desirable and have partly been accomplished in the case of VF scarring [12,13]. Taking into consideration the vibratory character of the VF, several models of dynamic bioreactors have been engineered and published. Many of these use an electromagnetic voice coil actuator to produce vibrational stimuli by moving cell-loaded scaffolds or cell culture plates with vibratory motion [4,5,14]. Yet, such motions considerably differ from actual vibratory movements of VF and can cause undesirable mechanical agitation.

We aimed to develop a device that fulfils three requirements: (1) to be composed mostly of commercially-available components to facilitate reproducibility, (2) cost-effectivenes, and (3) an wide range of frequencies and amplitudes for stimulation.

## Materials and methods

### Design and construction of the bioreactor

**Fig 1:**
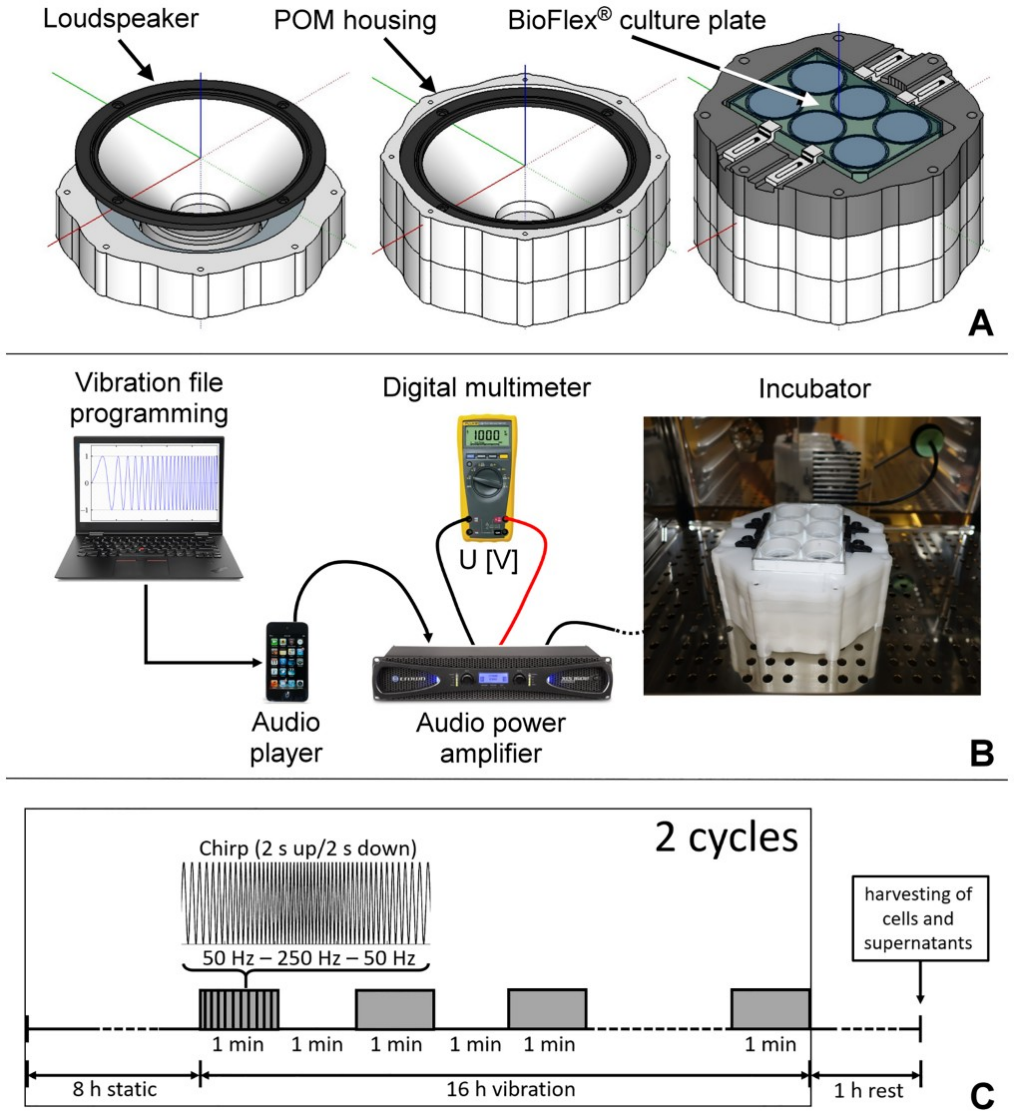
Construction of the bioreactor. A custom-made POM housing, designed using a 3D CAD software, accommodated the BioFlex plate (A). The schematic diagrams depict the assembly of all the components of the bioreactor (B) and the vibration pattern to which the cells were exposed to (C).

The vibration system consisted of a loudspeaker mounted into a custom-made polyoxymethylene (POM) housing with cell culture plate fixation elements (Fig 1A). The housing’s 3D model was designed by using the 3D computer-aided design (CAD) software SolidWorks (Dassault Systèmes SolidWorks Corp., Waltham, Massachusetts, USA) and was assembled from several computer numerical control (CNC) milled POM panels. Edges inside the housing were avoided as much as possible to prevent sound wave deflections. The loudspeaker (AL 170, 6.5" high-end low midrange driver, Visaton GmbH &Co.KG, Haan, Germany) had a nominal impedance of 8 Ω, a maximum power of 100 W and a mean sound pressure level of 88 dB. It was connected via an XLR audio cable to an audio power amplifier (XLS 1502, 775 W, Crown International, Elkhart, IN, USA) with an output power of 2x 300 W at 8 Ω. The audio cable was pulled through a sealed hole in the back of the incubator (ICO105, Memmert GmbH & Co. KG, Schwabach, Germany). We used 6-well-plates with a flexible 0.51 mm thick silicone base, with a surface area of 9.62 cm^2^ per well (BioFlex, Flexcell International Corporation, Burlington, NC, USA). These plates were developed for the Flexcell Tension System, a commercially available computer-controlled bioreactor that operates with vacuum and positive air pressure to move a cylindrical loading post that cyclically exerts strain to cells in the plates. However, this system only offers a maximum frequency of 5 Hz, and therefore could not meet our requirements for higher frequencies (50 Hz– 250 Hz) and more complex stimulation patterns. To overcome this limitation, we mounted the plates on the custom-made housing. This allowed us to stimulate the silicone membranes directly via sound waves and to couple any stimulation pattern with high amplitudes and frequencies up to 10 kHz.

The loudspeaker featured an anodised aluminium cone, a solid die-cast aluminium basket, an elastic rubber surround and a kapton voice-coil to withstand conditions in the cell culture incubator (37 °C, 100% humidity). The nearly linear frequency response between 50 Hz and 2500 Hz optimally covered our desired frequency range. The large cone displacement (+/- 11 mm) due to a long voice coil allowed the movement of a large volume of air, allowing for correspondingly large displacements of the silicone membranes. Stimulation sound files were generated with version 2.2.2 of the open source application for audio recording, editing and sound synthesizing software Audacity (audacityteam.org, registered trademark of Dominic Mazzoni) and were exported as 24 Bit/96 kHz audio files to a mobile audio player (iPod touch 4G, Apple Inc., Cupertino, CA, USA). The voltage at the audio output of the audio power amplifier served as a measure of the vibration intensity and was determined with a digital multimeter (Fluke 179, Fluke, Everett, WA, USA). The laboratory setting of the components is shown in Fig 1B.

### Vibration analysis

The vibration behavior of the silicone membranes in the culture plates was investigated by laser Doppler vibrometry (LDV) using a Polytec OFV 353 sensor head with a Polytec OFV 3001 vibrometer controller (Polytec, Waldbronn, Germany). Vibrational forces generated by the loudspeaker were transmitted to the flexible membranes of the culture plates via sound waves. The laser-optical measurement was carried out by the LDV 1mW laser beam focused onto and scanned over the vibrating surface. Merging this beam internally with the LDV reference beam enabled the detection of surface velocity and displacement amplitudes with nanometer resolution. A calibration constant of 125mm/s/V was used. A linear chirp signal (short frequency sweep from 1 Hz to 2500 Hz in 0.25 s) was used for acoustic broadband excitation of the membranes in order to record the natural resonance frequencies and vibration modes of the membranes. The LDV output voltage was sampled at 10 kHz sampling rate (SR) with a NI-9121 analog-digital input module and LabView 2012 (both National Instruments, Austin, TX, USA). A spectral analysis of the time signal was performed by a discrete Fourier transform (DFT) with the MatLab (The MathWorks, Inc., Natick, MA, USA) implemented pwelch algorithm using a rectangular window function and a sample length (SL) of 10000 samples, resulting in a 1Hz spectral resolution. The total sampling time was 60 s per position scanned, resulting in 60 spectra that were averaged. Even at a low signal-to-noise ratio (SNR) due to light scattering in the watery surface layer above the membrane, and assuming a 0.95 confidence interval, the error in amplitude per 1Hz bin was below 2%. From the LDV output voltage U_eff_ for each frequency bin in the spectrum, the displacement amplitude at that frequency was calculated using the calibration factor and the angular frequency 2πf, with f the frequency in Hz. Scanning the LDV probe volume along the surface provided the local oscillation amplitudes (local surface deflections) and thus the vibrational mode shapes at the resonance frequencies of the membrane. The measurement points were set at 3, 6, 9, 12, 15 and 17.5 mm radially from the center of the membrane. The measurements were carried out with and without medium in the wells.

### Cell culture

Immortalized human vocal fold fibroblasts (hVFF) [15], kindly provided by Prof. Susan Thibeault, University of Wisconsin-Madison, were cultured at 37°C with 5% CO_2_ in high glucose Dulbecco’s modified Eagle’s medium (DMEM, Sigma Aldrich,Vienna, AT) supplemented with 10% FBS (Biowest, Nuaillé, FR) and 100μg/ml Normocin (Invivogen, San Diego, CA, USA). The cells were trypsinized using 0.25% Trypsin/EDTA (Sigma Aldrich) and seeded on the flexible culture plates coated with pronectin at a density of 140 000 cells/well. Cells were allowed to attach for 24 hours in static conditions after which the medium was changed to DMEM containing 1% FBS, and the cells were transferred to the vibration bioreactor for 48 hours. Non-vibrational control cells were cultivated in parallel in a separate incubator. Cells were exposed to a vibration pattern as followed: eight hours without vibration (static) followed by 16 hours composed of one-minute vibration (linear chirp, range: 50 Hz-250 Hz-50 Hz) and one minute static; this pattern was repeated once more for a total duration of 48 hours. After a one-hour rest period, cells and supernatants were harvested for subsequent analyses (Fig 1C).

### Lactate dehydrogenase (LDH) assay

Quantification of cytotoxicity was performed with cell culture supernatants using the Pierce LDH Cytotoxicity Assay Kit (Thermo Fisher Scientific, Waltham, MA, USA). LDH release in the extracellular environment is one of the most commonly used ways of determining plasma membrane permeability caused by cell injury [16,17]. For each experiment, a maximum LDH activity control was run in parallel to the conditions of interest by seeding cells (with the same density) in a well of a 24-well plate. 45 min before sampling of supernatants, 10x lysis buffer was added to this well, followed by further incubation at 37 °C and 5% CO_2_. Subsequently, all samples were processed according to the manufacturer’s instructions. The LDH activity of the samples was expressed as percentage of the maximal LDH activity.

### Real time qPCR

Cells were harvested using the QIAZOL Lysis Reagent (Qiagen, Hilden, Germany) and total mRNA was isolated with the miRNeasy Mini Kit (Qiagen, Hilden, DE) according to the manufacturer’s instructions. Purified RNA was eluated in RNase-free water and concentration was determined using the NanoDrop 2000c spectrophotometer (Thermo Scientific). Reverse transcription (RT), as well as RT quantitative PCR (RT-qPCR), was performed as previously described [18]. Primer sequences are provided in Table 1. C_T_ values of technical triplicates were averaged and relative quantification of all mRNAs of interest was performed based on the the 2^-ΔΔC^_T_ method [19] with one modification: the geometric mean of the C_T_ values of B2M and UXT reference RNAs was used as internal normalization factor.

**Table 1:** Primer sequences used for RT-qPCR.

Gene	Gene symbol	Forward primer	Reverse primer	Product length [bp]
Alpha smooth muscle actin	ACTA2	CGTTACTACTGCTGAGCGTGA	GCCCATCAGGCAACTCGTAA	137
Beta-2-microglobulin	B2M	AGGCTATCCAGCGTACTCCA	CGGATGGATGAAACCCAGACA	105
Collagen I α1	COL1A1	CCCCGAGGCTCTGAAGGT	GCAATACCAGGAGCACCATTG	140
Collagen I α2	COL1A2	ACCACAGGGTGTTCAAGGTG	CAGGACCAGGGAGACCAAAC	149
Collagen III α1	COL3A1	GACCTGGAGAGCGAGGATTG	GTCCATCGAAGCCTCTGTGT	99
Fibronectin	FN1	CTGCAAGCCCATAGCTGAGA	GAAGTGCAAGTGATGCGTCC	147
Hyaluronan synthase 1	HAS1	CTTCCTAAGCAGCCTGCGAT	TATATAGGCCTAGAGGACCGCTG	104
Hyaluronan synthase 2	HAS2	ATGCTTGACCCAGCCTCATC	TTAAAATCTGGACATCTCCCCCAA	91
Hyaluronan synthase 3	HAS3	ATCATGCAGAAGTGGGGAGG	GAGTCGCACACCTGGATGTA	92
Hyaluronidase 1	HYAL1	AGCCTAGGTTGTCCTCGACC	GCATTCCAGACGGTGGTGAA	144
Hyaluronidase 2	HYAL2	CGTGGTCAATGTGTCCTGGG	CCCAGGACACATTGACCACG	81
Matrix metalloproteinase 1	MMP1	CACGCCAGATTTGCCAAGAG	GTTGTCCCGATGATCTCCCC	151
Transforming growth factor beta 1	TGFB1	TACCTGAACCCGTGTTGCTC	GCTGAGGTATCGCCAGGAAT	119
Ubiquitously expressed transcript protein	UXT	GCAGCGGGACTTGCGA	TAGCTTCCTGGAGTCGCTCA	104

### Western blotting

Cells were washed twice with ice-cold PBS and lysed in RIPA buffer (Cell Biolabs, San Diego, CA, USA) supplemented with 1x HaltProtease and Phosphatase Inhibitor Cocktail and 5mM EDTA (both Thermo Fisher Scientific). Protein content was determined using Pierce BCA Protein Assay Kit (Thermo Fisher Scientific) according to the manufacturer’s instructions. Twenty μg of total protein was mixed with appropriate amounts of 4x Laemmli Buffer (Bio-Rad, Hercules, CA, USA) and DTT and boiled for 5min at 95°C. SDS-PAGE was performed using 4–20% Mini PROTEAN TGX gels (Bio-Rad), after which the proteins were blotted onto a nitrocellulose membrane (Bio-Rad). The blots were blocked in 5% milk for two hours, followed by incubation with the primary antibody (FN, Proteintech Europe 1:10000; HAS2, Novus biological 1:1000; GAPDH, Cell signaling 1:5000) over night at 4°C. After washing, the blots were incubated with the appropriate HRP-conjugated secondary antibody. Bands were detected after the addition of SuperSignal West Pico Chemiluminescent Substrate (Thermo Fisher Scientific). Blot images were acquired with the ChemiDoc Touch system (Bio-Rad) and densitometric analysis was conducted using ImageLab software (Bio-Rad).

### Pepsin digestion, SDS-PAGE and silver stain

Analysis of fibrillar collagens was performed as described in a previous study [13]. Briefly, cells in 6-well plates were washed with PBS and incubated with digestion buffer (0.25 mg/mL Pepsin, 0.005% Triton X-100, 0.01% Phenol red in 250 mM HCl; 480 μL per well) for 2 h at room temperature with shaking (200 rpm). One milliliter of supernatant per well was collected and incubated with 100μL pepsin solution (1mg/mL pepsin in 1N HCl) and digested for 2 hours at room temperature with shaking (400rpm). Subsequently, pH was neutralized by addition of 1N NaOH (144 μL per well, 121 μL per supernatant tube). 18 μL of sample was mixed with 6 μL 4x Laemmli sample buffer and applied to 3–8% Criterion XT Tris-Acetate gels (Bio-Rad). VitroCol human type I collagen solution (Advanced BioMatrix) was used as standard and applied on a separate lane (0.16 μg in 18 μL ddH_2_O; pre-mixed with 6 μL Laemmli sample buffer (4x)). Electrophoresis was run at 200 V for 70 min. For fixation, the gel was placed in a solution of 40% EtOH and 10% acetic acid in ddH2O and incubated with agitation (50 rpm) at room temperature for 1h. Subsequently, the gel was stained using the SilverQuest Silver Staining kit (Thermo Scientific) according to the manufacturer’s protocol. Gel images were acquired with the ChemiDoc Touch system (Bio-Rad) and densitometric analysis was conducted using ImageLab software (Bio-Rad).

### Statistical analysis

All experiments were done at least three times in quadruplicates (RT-qPCR) or duplicates (western blot, silver stain). Statistical analysis was performed using Graph Pad Prism 7.0 software (Graph Pad, La Jolla, CA, USA). Student’s t-test was used to determine significant differences between the vibration conditions and static control. The results are presented as mean ± standard deviation (SD).

## Results

### Vibration analysis

**Fig 2:**
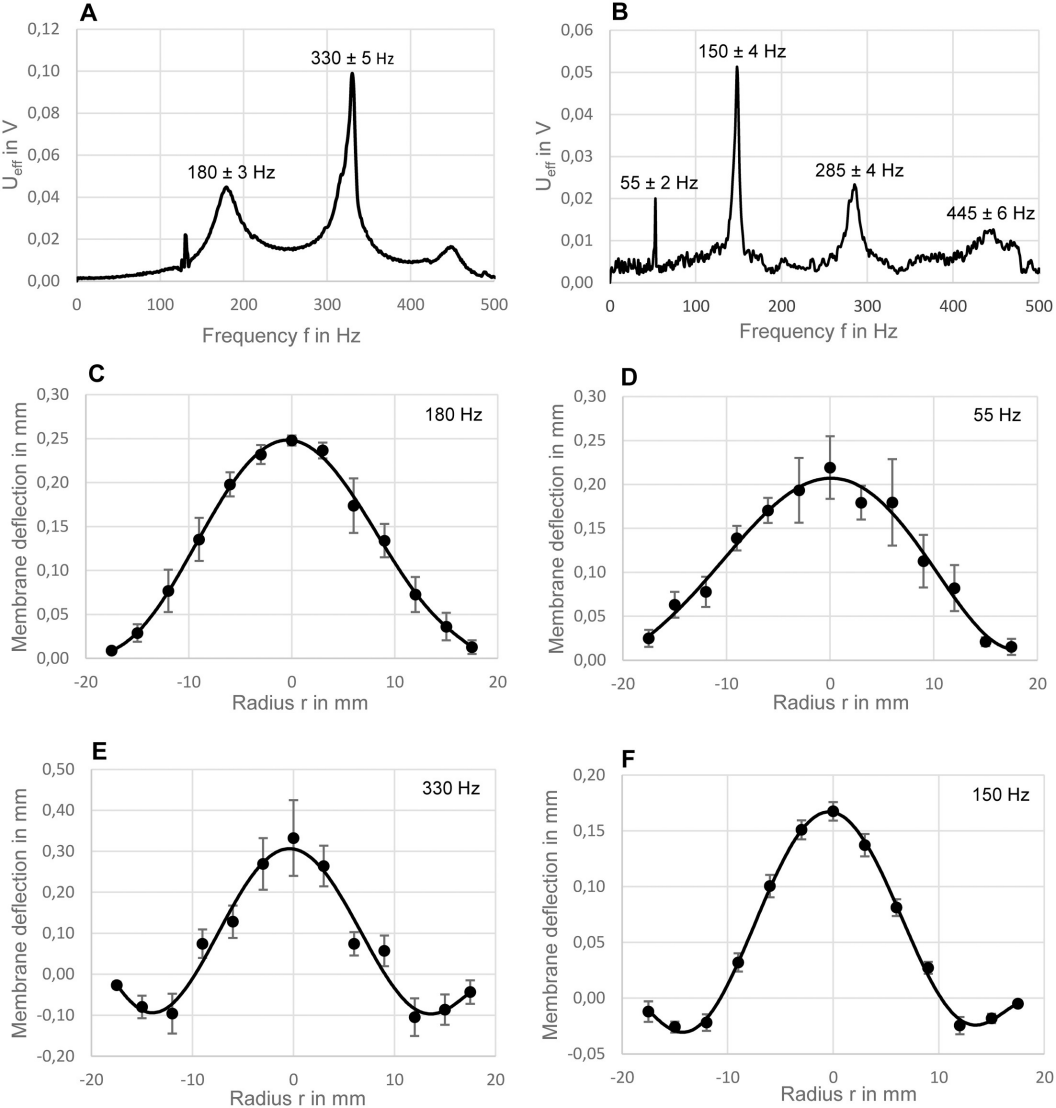
Laser Doppler vibrometry results. Vibration amplitude spectra and corresponding vibration modes recorded without medium in the wells (A, C, E) and with 2.5 ml medium (B, D, F). Frequency spectra (A, B) were plotted for the central position of one membrane. The fundamental modes of the membranes (C, D) (excitation frequency 180 Hz and 55Hz, respectively) and the first harmonic modes of the membranes (E, F) (excitation frequency 330 Hz and 150 Hz, respectively) were averaged values from all 6 membranes of one cell culture plate.

Laser Doppler vibrometry analysis of the silicone membranes was first performed without cell culture medium in the wells. The wells were then filled with 2.5 ml of medium, a typical volume used in cell culture for further measurements. The resulting oscillation amplitude spectra and corresponding vibration modes are shown in Fig 2. The excitation was elicited by sound waves, The mode shapes plotted were an average of all six membranes in one cell culture plate.

In order to identify the resonance frequencies of the oscillating membranes, the resulting oscillation amplitude spectra were plotted as root mean square values of the LDV voltage output signal (U_eff_) in Fig 2A and 2B for the central position of a single membrane. When testing the membranes without medium, the fundamental frequency was identified at 180 ± 3 Hz, the first harmonic at 310 ± 5 Hz (Fig 2A). With medium added, a shift towards lower frequencies was measured, with the fundamental frequency at 55 ± 2 Hz and the first harmonic at 150 ± 4 Hz. Higher harmonics were observed at 285±4 Hz and 445±6 Hz (Fig 2B). The small deviation in resonance frequency between the single membranes was likely to be caused by slightly different clamping and/or thickness of the membranes on the culture plate used.

The modal shape of circular membrane vibrations is represented by Bessel functions for the ideal axisymmetric case. Fig 2C–2F show the real mode shapes for the fundamental and first harmonic modes with and without medium in the well. The voltage data was converted to oscillation amplitudes (local deflections; see methods chapter “Vibration analysis”) and the final plots in Fig 2C–2F represent an average of all six membranes of one plate. The error bars indicate the variations in amplitude between the six membranes, as well as the accuracy in positioning of the sampling laser beam. For the first harmonic mode (Fig 2E and 2F) the largest strain was observed at a radial distance of approximately 3–9 mm from the center. By step-wise increasing the sound pressure and measuring the oscillation amplitude in the center of one membrane, a non-linear behavior of the membrane was found for displacement amplitudes above 1.5 mm (without medium) and 0.3 mm (55 Hz with 2.5 ml medium) (Table 2). This means that an increase in acoustic excitation pressure above these values no longer caused a corresponding increase in oscillation amplitudes. This non-linear stress-strain relation was quite dramatic and led to a droplet ejection from the surface observed at high oscillation amplitudes above 1 mm.

**Table 2:** Membrane deflections at different audio output voltages.

	Without medium (excitation 180 Hz)	With medium (excitation 55 Hz)
Voltage (V)	Membrane deflection (mm)	
**0.08**	0.063 ± 0.004	0.055 ± 0.011
**0.25**	0.195 ± 0.010	0.162 ± 0.082
**0.5**	0.405 ± 0,021	0.267 ± 0.066
**1**	0.864 ± 0.050	0.437 ± 0.071
**1.5**	1.316 ± 0.088	0.559 ± 0.090
**2**	1.691 ± 0.119	0.658 ± 0.041
**2.5**	1.950 ± 0.133	0.703 ± 0.030
**3**		0.759 ± 0.032
**3.3**		0.785 ± 0.045

### Cell viability

**Fig 3:**
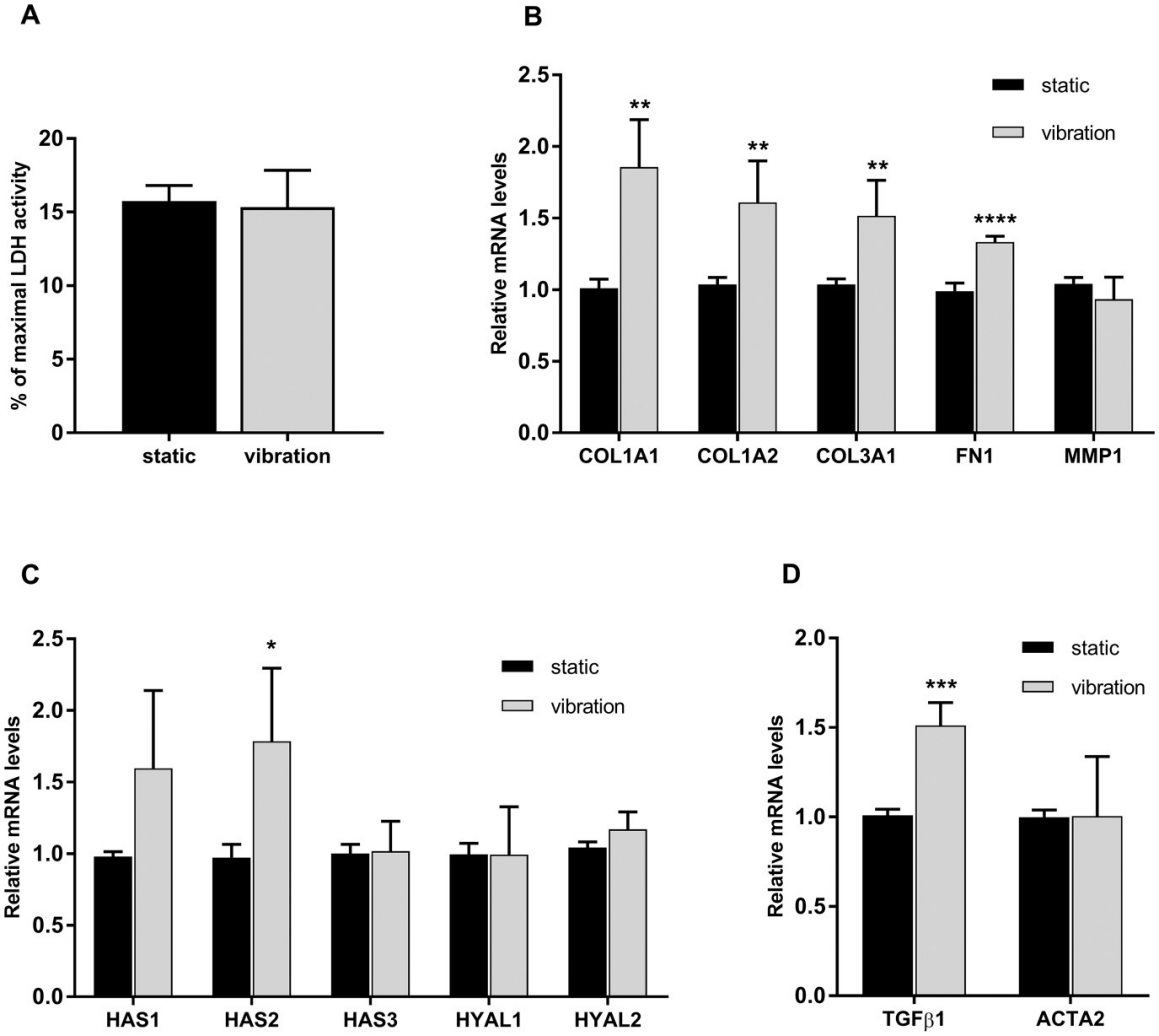
Effect of vibration on cell viability and gene expression. After 48 hours of exposure to a vibration pattern and a one-hour rest period, supernatants and cell lysates were collected for LDH activity assay (A) and RNA isolation, respectively. mRNA was reverse transcribed to cDNA, and RT-qPCR was performed. Relative gene expression (B, C, D) was calculated using the 2^-ΔΔC^_T_ method. The values are mean ± SD of at least 3 experiments performed in quadruplicates and analysed by a Student’s t-test; *p<0.05,**p<0.01,***p<0.0001.

Lactate dehydrogenase (LDH) activity was used to measure the effects of vibration on cell viability. As shown in Fig 3A, LDH activity assay showed no difference between static control cells and cells exposed to vibration.

### Gene expression and protein synthesis

We examined the effects of vibration on several extracellular matrix—related genes using RT-qPCR. We found that, in our experimental setup, the vibration significantly induced the gene expression of collagens: COL1A1, COL1A2, COL3A1, fibronectin (Fig 3B) and hyaluronan synthase 2 (HAS2) (Fig 3C), compared to static control cells.

Other ECM-related genes showed no statistically significant change. We also investigated whether the vibration caused a shift to a pro-fibrotic myofibroblast phenotype, and although the vibration significantly increased the expression of TGFβ1, the expression of ACTA2, a marker of myofibroblasts remained unchanged compared to static control cells (Fig 3D).

**Fig 4:**
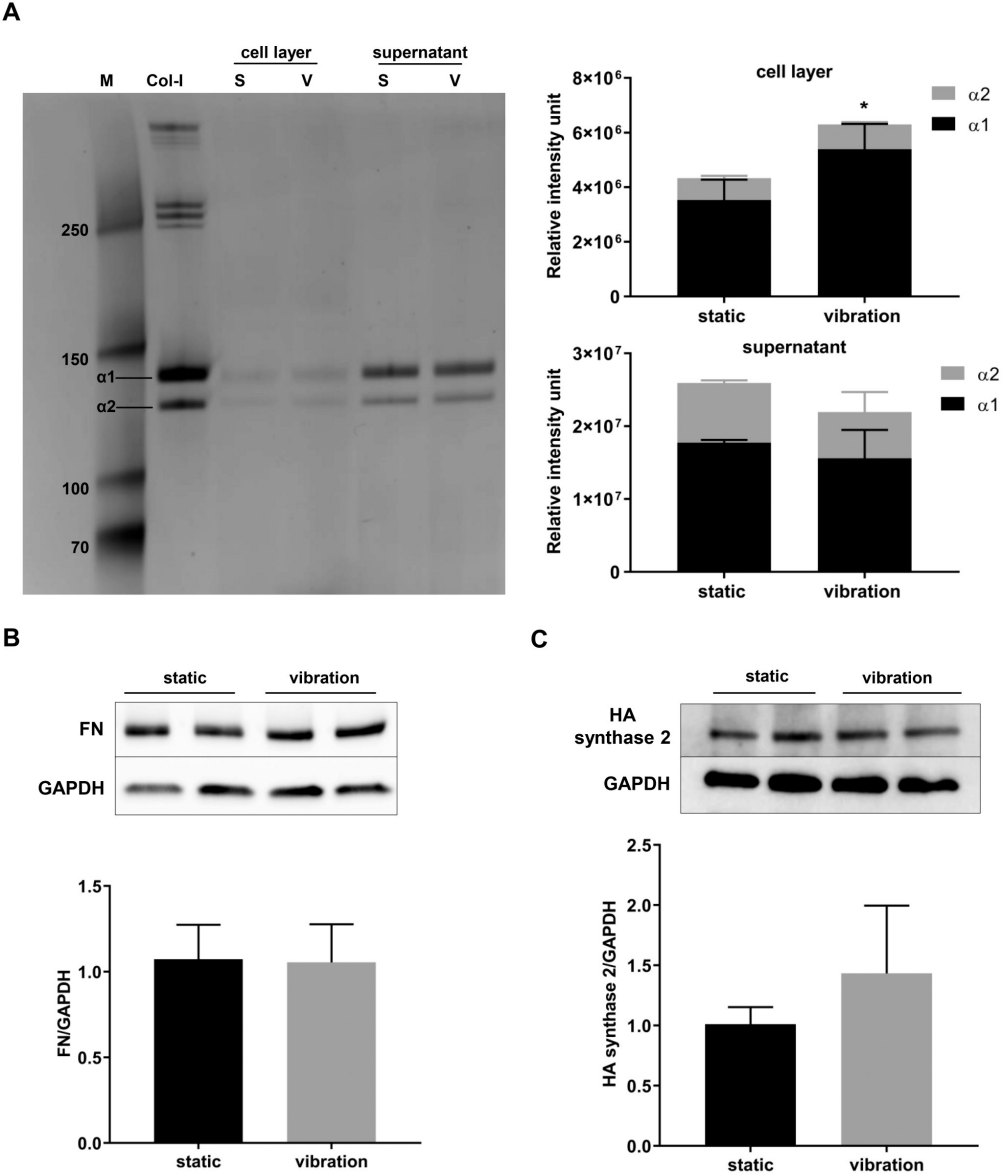
Effect of vibration on ECM-related protein synthesis. Supernatants and cell layers were digested with pepsin for two hours, or cells layers were lysed with RIPA buffer. Pepsin digested samples were subjected to SDS-PAGE and proteins were stained with silver (A). Proteins isolated in RIPA buffer were subjected to SDS-PAGE and Western blot, after which fibronectin (B) and HA synthase 2 (C) were detected with specific antibodies. GAPDH was used as a loading control. The values are mean ± SD of three experiments performed in duplicates and analysed by a Student’s t-test; *p<0.05.

Collagen I protein content was examined in the cell layers and supernatants after digesting the samples with pepsin. Silver stain showed α1 and α2 bands in cell layers and supernatants that corresponded to collagen I α1 (139 kDa) and collagen I α2 (129 kDa) proteins, respectively. Collagen-1 α1 production was increased after vibration in the cell layer, but not the supernatant (Fig 4A). We found no effect of vibration on the protein synthesis of fibronectin and HA synthase 2 (Fig 4B and 4C).

## Discussion

To advance the understanding of numerous VF diseases as well as tissue-engineering based treatment options, a newer generation of dynamic bioreactors is required to more closely reproduce the *in vivo* conditions of VFF. Recently, several devices were engineered and published [4,5,14,20–23]. However, many of these devices are technically highly sophisticated, and hence very costly, making it difficult for other groups to reconstruct. The aim of our project was to engineer and validate a dynamic bioreactor based on commercially-available components that is cost effective, easy to assemble and offers a wide range of phonatory stimulation patterns simulating the wide spectrum of the human voice.

The bioreactor closest to the complex structure of the human larynx so far, is the flow perfusion bioreactor developed by Latifi et al. [20]. They designed two VF replicas in which human VFF were seeded, that were set in vibration by using an air-blower beneath the constructed bioreactor. The limitation of this bioreactor was its frequency span, as the tested frequency ranged from 0.5 to approximately 100 Hz, which includes only the male fundamental frequency. To overcome the limitations of mechanically-induced vibration, Farran et al. constructed a bioreactor that transferred the vibration to the cells via acoustic wave propagation [21]. However, this bioreactor had some limitations, as acknowledged by the authors, for instance, the inability to accommodate the vibration stage containing cells to a standard microscope for *in situ* visualisation. Kim et al. designed a bioreactor where commercially-available cell culture plates with flexible silicone bottoms were used for easy visualisation and subsequent analyses of the cultured cells, yet the vibration was transferred mechanically through small linear actuators [22]. This limits the frequency range to which the cells could be exposed. Our bioreactor combines the practicality of standardized, commercially-available cell culture plates with flexibility of using sound wave propagation as a source of vibration. Sound wave propagation with a frequency range from 50–2500 Hz, enables the use of different stimulation patterns and frequencies. Due to the design of the bioreactor, the loudspeaker propagates sound waves directly below the cell culture plate, simultaneously transferring the vibration to all six wells of the plate. The novelty of our bioreactor is the use of custom sound files, which makes it possible to change vibration frequency and pattern during a single experiment without the physical presence of the experimenter.

We decided to use a chirp sound pattern (50 Hz -250 Hz-50 Hz) in order to create membrane movements where the majority of the cells would be exposed to membrane deformation and thus tensile and shear stress. The chosen frequency span also included the fundamental frequencies of human voice (120 Hz (male) to 220 Hz (female)).

In our study, VFF exposed to vibration showed a significant change in ECM-related gene expression compared to static control. Hyaluronic acid (HA) and collagen are major components of the VF *lamina propria*. HA provides tissue viscosity and affects tissue flow resistance [24], while collagens provide structural strenght to tissue and are crucial in resisting stress and deformation, when subjected to force [2]. The distribution and quantity of *lamina propria* components is important as it has a direct effect on phonation. We found that the gene expression of HAS2, a hyaluronan synthase responsible for the synthesis of high molecular weight HA [25], was significantly upregulated (1.7- fold, compared to control) in cells exposed to vibration. This is in line with previous studies from Titze and colleagues [4] and Kutty and Webb [5], that showed a 2.5-fold and 4.5-fold gene upregulation, respectively. The mentioned studies however, used cells incorporated in a three-dimensional substrate, and the exposure of cells to vibration differed from our conditions (6 hours and 10 days, respectively). One major difference to the results published by Kutty and Webb [5] is their use of human dermal fibroblasts. We found no change in the protein levels of HA synthase 2 after vibration. To the best of our knowledge, other publications have not analysed HA synthase 2 protein synthesis after vibration. Collagens type I and III were also significantly upregulated compared to static control cells (1.8-fold and 1.5-fold, respectively). It is known that collagen type I expression differs under dynamic culture conditions and is strongly dependent on the frequency and amplitude [21]. This may explain the differences in collagen gene expression (up- or downregulation) seen in previous dynamic culture studies [4,5,21–23]. A summary of the effects of vibration on collagen type I gene expression and other biological effects from cited studies is listed in Table 3. We found that protein levels of collagen type I, α1 were increased 1.5-fold after vibration. Latifi et al. [20] found a 5-fold increase in collagen type I protein levels. The results are not directly comparable to ours, as the cells were grown in a three-dimensional hydrogel matrix.

**Table 3:** Summary table of the vocal fold bioreactors cited in the article.

**Publication**	Titze et al. [4]	Wolchok et al. [14]	Kutty and Webb [5]	Gaston et al. [25]	Farran et al. [21]	Latifi et al. [20]	Kim et al. [22]	Present study
**Reactor type**	3D-axial and vibratory stimulation	3D-substrate vibratory stimulation	3D-vibratory stimulation	3D-axial and vibratory stimulation	2D-electro-acoustically driven	3D-perfusion phonation-induced stimulation	2D- vibratory stimulation	2D- electro acoustically driven
**Source of vibration**	voice coil actuator	voice coil actuator	voice coil actuator	voice coil actuator	loudspeaker	variable speed centrifugal air blower	linear actuator	loudspeaker
**Cell type**	Human laryngeal fibroblasts	Human laryngeal fibroblasts	Human dermal fibroblasts	Human vocal fold fibroblasts, bone marrow mesenchymal stem cells	Neonatal foreskin fibroblasts	Human vocal fold fibroblasts	Human vocal fold fibroblasts	Human vocal fold fibroblasts
**Culture substrate**	Tecoflex substrate	Tecoflex substrate	Methacrylated hyaluronate hydrogel	Fibronectin coated Tecoflex substrate	Collagen-I coated silicone membranes	HA-Ge hydrogel	Collagen-I coated Bioflex plate	Pronectin coated Bioflex plate
**Duration of experiment**	6 hours	3 days /21 days	1/3/5/10 days	1 day	1 day	2 days	2/6/10 hours	2days
**Stimulation/ vibration frequency**	20% axial strain/ 100 Hz	100 Hz	100 Hz	20% axial strain/ 200 Hz	60/110/300 Hz	~100 Hz	205 Hz	50-250Hz
**Stimulation per day**	6 hours	6 hours	2 hours	8 hours	1 hour	2 hours	2/6/10 hours	8h
**Stimulation pattern**	continuous	1 s vibration/ 2 s static	2 s vibration/2 s static	continuous	continuous	1 h phonation/15 min rest/1 h phonation	continuous	1 min vibration/1 min static for 16 hours
**tested frequency range of reactor**	20–200 Hz	100–200 Hz	not discussed	0–2727 Hz	0–400 Hz	0,5–100 Hz	not discussed	50–2500 Hz
**Biological effects of vibration compared to static conditions**								
**collagen- I**	1.5-fold gene upregulation	1.7-fold increased protein expression	20% reduction of total collagen protein (d5)	no effect	1.2-fold gene upregulation (60 Hz), 0.75-fold downregulation (110 Hz)	5-fold increased protein expression	no effect	1.8-fold gene upregulation, 1.5-fold increased protein expression
**collagen-III**	NA	NA		NA	NA	2.4-fold increased protein expression	NA	1.5-fold gene upregulation
**HA synthase 2**	2.5-fold gene upregulation	NA	5-fold gene upregulation (d3)	NA	NA	NA	10–20% gene downregulation	1.6-fold gene upregulation
**fibronectin**	~2.3-fold gene upregulation	~2-fold increased protein expression	NA	no effect	no effect	NA	no effect	1.3-fold gene upregulation
**MMP1**	~3-fold gene upregulation	NA	2-fold gene upregulation (d5)	NA	10% gene downregulation 60 Hz	NA	no effect	no effect
**TGF-β1**	NA	2 -fold increased protein expression in medium	NA	no effect	NA	NA	NA	1.5-fold gene upregulation

NA-not analysed

The expression of fibronectin, a scaffolding protein involved in maintenance and organisation of the ECM [26], was slightly, but significantly upregulated in our conditions. Protein levels of fibronectin were not altered after vibration. Previous studies using 3D scaffolds found that vibration upregulated fibronectin gene [4] and protein expression [14].

Heavy voice use, causing continuous traumatic events, leads to a deterioration of VF tissue, which initiates a cascade of biochemical events that ultimately results in a remodelling of functional tissue [27]. TGFβ1 is a cytokine known to induce differentiation of fibroblasts to myofibroblasts which aid in wound contraction, yet prolonged TGFβ1 stimulation leads to excessive collagen production that causes fibrosis and VF scarring [28]. We found TGFβ1 gene expression to be significantly upregulated (1.5-fold) in cells exposed to vibration. However, ACTA2, a marker of myofibroblasts, was not changed compared to control cells. In a previous study, Gaston et al. exposed VFF to a total of eight hours of vibration, and saw no upregulation of TGFβ1 expression [23]. In our setting, cells were exposed to eight hours of vibration per day for two days, which may indicate that TGFβ1 gene expression is only induced after longer periods of voice use. Even though cells were exposed to vibration for a longer period of time than typical for heavy voice users [29], it did not affect cell viability.

Direct comparison with previously published bioreactors is difficult due to differences in the source of vibration, frequency, cell type and substrate in which the cells were cultured (Table 3).

However, when compared to two other 2D bioreactors discussed [21,22], our bioreactor conditions led to a higher level of gene upregulation of COL1A1 (1.8-fold increase compared to 1.2-fold and no effect, respectively). HAS 2 expression was not analysed by Farran et al.[21], while Kim et al. [22] found a 10–20% reduction in gene expression (1.6-fold induction by our bioreactor).

Although our device is simple in its construction, the biological effects on the cultured cells are similar to the ones achieved in much more sophisticated bioreactors [4,5,20].

We are aware that the limiting factor of this bioreactor is its two-dimensional nature, as cells in the VF find themselves in a much more complex, three-dimensional environment. Our future work will focus on finding appropriate 3D-scaffold matrices that could host cells and be able to vibrate within the range of the human voice. Introducing cell types other than fibroblasts (e.g. epithelial cells, macrophages) is another goal since the human VFs contain several cell types. This will pave the way to a new, more comprehensive *in vitro* VF model, which will allow studying cell interactions, response to injury and delivery of drugs.

## Supporting information

S1 Fig Representative western blot and silver stain images of the whole membrane and gel, respectively. HA synthase 2 WB (A), fibronectin WB (B) and collagen type I silver stain (C). GAPDH from the HA synthase 2 WB was detected on the same membrane after stripping. M-marker; S-static; V-vibration; c-cell layer; s-supernatant; Col I- collagen type I.
(TIF)

S1 File Excel spreadsheet containing raw data from the study. Each sheet contains the individual data points used in a particular figure, as noted.
(XLSX)

## References

[1] Catten M, Gray SD, Hammond TH, Zhou R, Hammond E. Analysis of cellular location and concentration in vocal fold lamina propria. Otolaryngol Head Neck Surg. 1998 5;118(5):663–7. 10.1177/019459989811800516 9591866

[2] Gray SD. Cellular physiology of the vocal folds. Otolaryngol Clin North Am. 2000 8;33(4):679–98. 10918654 10.1016/s0030-6665(05)70237-1

[3] Li NYK, Heris HK, Mongeau L. Current Understanding and Future Directions for Vocal Fold Mechanobiology. J Cytol Mol Biol. 2013 4 1;1(1):001 10.13188/2325-4653.1000001 24812638 PMC4011392

[4] Titze IR, Hitchcock RW, Broadhead K, Webb K, Li W, Gray SD, et al Design and validation of a bioreactor for engineering vocal fold tissues under combined tensile and vibrational stresses. J Biomech. 2004 10;37(10):1521–9. 10.1016/j.jbiomech.2004.01.007 15336927

[5] Kutty JK, Webb K. Vibration stimulates vocal mucosa-like matrix expression by hydrogel-encapsulated fibroblasts. J Tissue Eng Regen Med. 2010 1;4(1):62–72. 10.1002/term.219 19842110 PMC2849844

[6] Branski RC, Perera P, Verdolini K, Rosen CA, Hebda PA, Agarwal S. Dynamic biomechanical strain inhibits IL-1beta-induced inflammation in vocal fold fibroblasts. J Voice. 2007 11;21(6):651–60. 10.1016/j.jvoice.2006.06.005 16905293 PMC4948979

[7] Gugatschka M, Ainödhofer H, Gruber H-J, Graupp M, Kieslinger P, Kiesler K, et al Age effects on extracellular matrix production of vocal fold scar fibroblasts in rats. Eur Arch Otorhinolaryngol. 2014 5;271(5):1107–12. 10.1007/s00405-013-2722-7 24077847

[8] Krischke S, Weigelt S, Hoppe U, Köllner V, Klotz M, Eysholdt U, et al Quality of life in dysphonic patients. J Voice. 2005 3;19(1):132–7. 10.1016/j.jvoice.2004.01.007 15766858

[9] Gugatschka M, Ohno S, Saxena A, Hirano S. Regenerative medicine of the larynx. Where are we today? A review. J Voice. 2012 9;26(5):670.e7–13. 22795981 10.1016/j.jvoice.2012.03.009

[10] Ling C, Li Q, Brown ME, Kishimoto Y, Toya Y, Devine EE, et al Bioengineered vocal fold mucosa for voice restoration. Sci Transl Med. 2015 11 18;7(314):314ra187 10.1126/scitranslmed.aab4014 26582902 PMC4669060

[11] Ishii K, Yamashita K, Akita M, Hirose H. Age-related development of the arrangement of connective tissue fibers in the lamina propria of the human vocal fold. Ann Otol Rhinol Laryngol. 2000 11;109(11):1055–64. 10.1177/000348940010901112 11089998

[12] Graupp M, Gruber H-J, Weiss G, Kiesler K, Bachna-Rotter S, Friedrich G, et al Establishing principles of macromolecular crowding for in vitro fibrosis research of the vocal fold lamina propria. Laryngoscope. 2015 6;125(6):E203–209. 10.1002/lary.25103 25545625

[13] Graupp M, Rinner B, Frisch MT, Weiss G, Fuchs J, Sundl M, et al Towards an in vitro fibrogenesis model of human vocal fold scarring. Eur Arch Otorhinolaryngol. 2018 5;275(5):1211–8. 10.1007/s00405-018-4922-7 29520499 PMC5893733

[14] Wolchok JC, Brokopp C, Underwood CJ, Tresco PA. The effect of bioreactor induced vibrational stimulation on extracellular matrix production from human derived fibroblasts. Biomaterials. 2009 1;30(3):327–35. 10.1016/j.biomaterials.2008.08.035 18937972

[15] Chen X, Thibeault SL. Novel isolation and biochemical characterization of immortalized fibroblasts for tissue engineering vocal fold lamina propria. Tissue Eng Part C Methods. 2009 6;15(2):201–12. 10.1089/ten.tec.2008.0390 19108681 PMC2819707

[16] Danpure CJ. Lactate dehydrogenase and cell injury. Cell Biochemistry and Function. 1984 7;2(3):144–8. 10.1002/cbf.290020305 6383650

[17] Kumar P, Nagarajan A, Uchil PD. Analysis of Cell Viability by the Lactate Dehydrogenase Assay. Cold Spring Harbor Protocols. 2018 6;2018(6):pdb.prot095497. 10.1101/pdb.prot095497 29858337

[18] Karbiener M, Darnhofer B, Frisch M-T, Rinner B, Birner-Gruenberger R, Gugatschka M. Comparative proteomics of paired vocal fold and oral mucosa fibroblasts. J Proteomics. 2017 23;155:11–21. 10.1016/j.jprot.2017.01.010 28099887 PMC5389448

[19] Livak KJ, Schmittgen TD. Analysis of relative gene expression data using real-time quantitative PCR and the 2(-Delta Delta C(T)) Method. Methods. 2001 12;25(4):402–8. 10.1006/meth.2001.1262 11846609

[20] Latifi N, Heris HK, Thomson SL, Taher R, Kazemirad S, Sheibani S, et al A Flow Perfusion Bioreactor System for Vocal Fold Tissue Engineering Applications. Tissue Eng Part C Methods. 2016;22(9):823–38. 10.1089/ten.tec.2016.0053 27537192 PMC5035918

[21] Farran AJE, Teller SS, Jia F, Clifton RJ, Duncan RL, Jia X. Design and characterization of a dynamic vibrational culture system. J Tissue Eng Regen Med. 2013 3;7(3):213–25. 10.1002/term.514 22095782 PMC4076702

[22] Kim D, Lim J-Y, Kwon S. Development of Vibrational Culture Model Mimicking Vocal Fold Tissues. Ann Biomed Eng. 2016;44(10):3136–43. 10.1007/s10439-016-1587-5 26951463

[23] Gaston J, Quinchia Rios B, Bartlett R, Berchtold C, Thibeault SL. The response of vocal fold fibroblasts and mesenchymal stromal cells to vibration. PLoS ONE. 2012;7(2):e30965 10.1371/journal.pone.0030965 22359557 PMC3281043

[24] Gray SD, Titze IR, Chan R, Hammond TH. Vocal fold proteoglycans and their influence on biomechanics. Laryngoscope. 1999 6;109(6):845–54. 10369269 10.1097/00005537-199906000-00001

[25] Itano N, Sawai T, Yoshida M, Lenas P, Yamada Y, Imagawa M, et al Three isoforms of mammalian hyaluronan synthases have distinct enzymatic properties. J Biol Chem. 1999 8 27;274(35):25085–92. 10455188 10.1074/jbc.274.35.25085

[26] To WS, Midwood KS. Plasma and cellular fibronectin: distinct and independent functions during tissue repair. Fibrogenesis Tissue Repair. 2011 9 16;4:21 10.1186/1755-1536-4-21 21923916 PMC3182887

[27] Verdolini Abbott K, Li NYK, Branski RC, Rosen CA, Grillo E, Steinhauer K, et al Vocal exercise may attenuate acute vocal fold inflammation. J Voice. 2012 11;26(6):814.e1–13. 10.1016/j.jvoice.2012.03.008 PMC3509805 23177745

[28] Mia MM, Boersema M, Bank RA. Interleukin-1β attenuates myofibroblast formation and extracellular matrix production in dermal and lung fibroblasts exposed to transforming growth factor-β1. PLoS ONE. 2014;9(3):e91559 10.1371/journal.pone.0091559 24622053 PMC3951452

[29] Hunter EJ, Titze IR. Variations in intensity, fundamental frequency, and voicing for teachers in occupational versus nonoccupational settings. J Speech Lang Hear Res. 2010 8;53(4):862–75. 10.1044/1092-4388(2009/09-0040) 20689046 PMC3302664

