# Supplementary figures and images for: Development and validation of a novel phonomimetic bioreactor
Source: PLoS One. 2019 Mar 14;14(3):e0213788. doi: 10.1371/journal.pone.0213788 (PMC6417646; doi:10.1371/journal.pone.0213788)

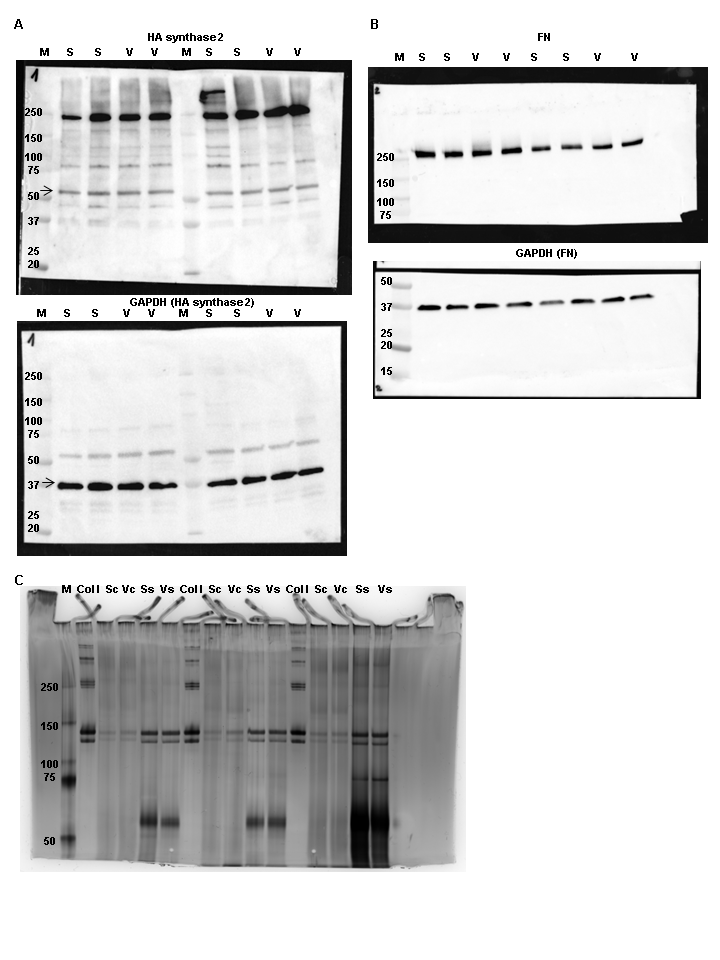

Supplement: S1 Fig — HA synthase 2 WB (A), fibronectin WB (B) and collagen type I silver stain (C). GAPDH from the HA synthase 2 WB was detected on the same membrane after stripping. M-marker; S-static; V-vibration; c-cell layer; s-supernatant; Col I- collagen type I. (TIF) [file pone.0213788.s001.tif]
